# Advanced Membranes and Membrane Technologies for Wastewater Treatment

**DOI:** 10.3390/membranes16070233

**Published:** 2026-07-03

**Authors:** Juan L. Acero, Francisco J. Real

**Affiliations:** Departamento de Ingeniería Química y Química Física, Instituto Universitario de Investigación del Agua, Cambio Climático y Sostenibilidad (IACYS), Universidad de Extremadura, Avenida de Elvas s/n, 06006 Badajoz, Spain

## 1. Introduction

The increasing demand for clean water, rapid industrialization, urban population growth, and stricter environmental regulations have intensified the need for efficient and sustainable wastewater treatment technologies [1,2]. Wastewater generated from municipal, industrial, and agricultural sources contains a wide range of contaminants, and the discharge of untreated or inadequately treated wastewater poses significant risks to human health, aquatic ecosystems, and water resources. Among the various treatment technologies available, membrane-based processes have emerged as highly effective and versatile methods for wastewater treatment and water reclamation [3].

Recent advancements in membrane science have led to the development of novel membrane materials, surface modification techniques, nanocomposite membranes, and hybrid treatment systems that significantly improve permeability, selectivity, fouling resistance, and operational stability [4,5,6]. The combination of membrane processes with biological, chemical, and electrochemical technologies has enabled more efficient removal of recalcitrant pollutants while reducing energy consumption and operational costs [7]. Despite these advancements, several challenges continue to limit the widespread implementation of membrane technologies, including membrane fouling, scaling, high energy requirements, membrane degradation, and concentrate disposal issues [4]. Consequently, ongoing research focuses on developing more sustainable, cost-effective, and energy-efficient membrane systems capable of meeting the growing demand for wastewater reuse and resource recovery.

This Special Issue features nine compelling papers that are intended to cover recent developments related to membrane materials, membrane fouling prediction, and urban and industrial wastewater treatment by advanced membrane processes, while discussing current challenges, emerging trends, and prospects for enhancing the efficiency and sustainability of wastewater treatment processes.

## 2. Overview of Special Issue Contributions

In this collection are two review articles that provide comprehensive perspectives on membrane-based technologies for water treatment. Dashtban Kenari et al. (Contribution 1) present a critical review of ceramic membrane technologies for the treatment of mining effluents. Owing to their excellent chemical stability, mechanical strength, and resistance to harsh operating conditions, ceramic membranes have gained increasing attention for industrial wastewater treatment. The review covers the application of several membrane processes, including microfiltration (MF), ultrafiltration (UF), nanofiltration (NF), and vacuum membrane distillation (VMD). The authors emphasized the need for future research to investigate ceramic membranes with diverse pore structures, either as standalone systems or integrated with complementary treatment technologies, with particular attention paid to process water recycling in mineral processing operations. Similarly, Xun et al. (Contribution 2) reviewed the current state of nitrogen removal in membrane bioreactor (MBR) systems, highlighting recent advances in the understanding of the underlying removal mechanisms. Their work systematically evaluates the environmental parameters governing nitrogen removal efficiency and discusses emerging MBR-based configurations designed to enhance nutrient recovery and energy efficiency. The review concludes that continued technological improvements could enable MBR systems to evolve into sustainable, resource-oriented platforms, facilitating the transformation of conventional wastewater treatment plants into energy-positive and resource-recovering facilities.

Several original research articles included in this Special Issue focus on advancing conventional membrane technologies for urban wastewater treatment, with particular emphasis on the removal of emerging micropollutants. Ezeogu et al. (Contribution 3) compared the predictive performance of Response Surface Methodology (RSM) and Artificial Neural Network (ANN) models for estimating the rejection efficiencies of caffeine and paracetamol during nanofiltration. The latter demonstrated superior predictive accuracy, highlighting its potential as a robust optimization tool for membrane-based pharmaceutical removal. Aldana et al. (Contribution 4) evaluated the removal of eleven representative micropollutants from municipal wastewater using UF and NF membranes, while also investigating the effects of coagulation and powdered activated carbon (PAC) pretreatment. Both pretreatment strategies effectively mitigated membrane fouling and enhanced the removal of organic and inorganic contaminants; furthermore, PAC pretreatment significantly improved micropollutant retention in the integrated PAC/UF process, indicating that both standalone NF and combined PAC/UF systems represent viable alternatives for producing reclaimed water suitable for diverse reuse applications. Rodríguez-Sáez et al. (Contribution 5) investigated the application of recycled ultrafiltration (r-UF) membranes in aerobic MBR systems. Both recycled and modified recycled membranes achieved high permeate quality while exhibiting lower fouling propensity than commercial membranes operated under comparable conditions. These findings demonstrate that recycled UF membranes constitute a sustainable and economically attractive alternative for urban wastewater treatment, offering reduced membrane replacement requirements while maintaining treatment performance.

This collection also includes several contributions addressing recent developments in membrane technologies for industrial wastewater treatment. Lee et al. (Contribution 6) investigated fouling control in a submerged anaerobic ceramic membrane bioreactor (AnCMBR) treating high-strength food-processing wastewater. The system maintained stable long-term operation, effective fouling control, and high treatment efficiency under elevated organic loading conditions, demonstrating its suitability for decentralized treatment applications. Adu et al. (Contribution 7) evaluated an integrated treatment system combining a membrane-aerated biological reactor (MABR) with brackish water reverse osmosis (BWRO) for the treatment of humidity condensate generated in space habitation systems. Their results indicate that this hybrid process could substantially improve water recovery and operational reliability in extraterrestrial habitats while also offering potential applications in remote, isolated, and water-scarce terrestrial environments. In the field of produced water treatment, Madduri et al. (Contribution 8) developed a dual-layer zwitterion-modified electrospun nanofibrous forward osmosis (FO) membrane. Surface zwitterion functionalization enhanced membrane hydration, thereby reducing organic and biological fouling while simultaneously improving water transport properties, including higher water flux, excellent solute rejection, reduced concentration polarization, and superior antifouling performance relative to conventional FO membranes. These characteristics highlight the membrane’s potential for large-scale produced water treatment and reuse. Finally, Kempa et al. (Contribution 9) demonstrated the application of tubular polymeric UF membranes in a closed-loop water recycling system within a rubber manufacturing facility. The membranes reduced wastewater turbidity by more than 95% while achieving high separation efficiencies for the target contaminants, confirming their suitability for industrial water reclamation and circular water management.

## 3. Conclusions

In summary, this Special Issue presents a comprehensive collection of studies highlighting recent advances in membrane-based technologies for the treatment of urban and industrial wastewater. Collectively, these contributions demonstrate that membrane processes have evolved from complementary treatment options to core technologies within modern water treatment systems. The integration of advanced environmentally sustainable materials with green membrane fabrication strategies represents a significant step toward developing high-performance membranes that combine enhanced separation efficiency, operational robustness, and adaptability to diverse industrial applications while minimizing environmental impacts. Furthermore, the optimization and integration of membrane-based treatment processes are essential to ensuring the production of high-quality reclaimed water, mitigating chemical and biological contaminants, and improving the sustainability and efficiency of industrial operations. Continued research focused on the rational design of membrane materials, process intensification, fouling mitigation, and large-scale implementation will be critical to overcoming current technological limitations and enabling the widespread adoption of these systems. It is anticipated that the findings presented in this Special Issue will not only provide an up-to-date overview of membrane applications in wastewater treatment but also stimulate further scientific innovation and technological development toward sustainable global water management.
